# Preoperative BOLD cerebrovascular reactivity correlates with intraoperative STA-MCA bypass flow and influences postoperative CVR improvement

**DOI:** 10.1177/23969873251337234

**Published:** 2025-05-10

**Authors:** Martina Sebök, Vittorio Stumpo, Jacopo Bellomo, Giuseppe Esposito, Christiaan Hendrik Bas van Niftrik, Zsolt Kulcsár, Andreas R. Luft, Luca Regli, Jorn Fierstra

**Affiliations:** 1 Department of Neurosurgery, University Hospital Zurich, University of Zurich, Zurich, Switzerland; 2 Clinical Neuroscience Center, University Hospital Zurich, Zurich, Switzerland; 3 Department of Neuroradiology, University Hospital Zurich, University of Zurich, Zurich, Switzerland; 4 Department of Neurology, University Hospital Zurich, University of Zurich, Zurich, Switzerland

**Keywords:** BOLD MRI, cerebrovascular reactivity, revascularization, STA-MCA bypass, intraoperative flow

## Abstract

**Introduction:**

The superficial temporal artery-middle cerebral artery (STA-MCA) flow augmentation bypass is effective for treating Moyamoya vasculopathy and selected cases of atherosclerotic large vessel disease. Recently, blood oxygen level-dependent cerebrovascular reactivity (BOLD-CVR) has emerged as a novel tool to assess hemodynamic impairment for patient selection and monitoring. This study investigates whether preoperative BOLD-CVR in the affected vascular territory (i.e. middle cerebral artery (MCA) territory) correlates with intraoperative bypass flow and whether intraoperative bypass flow serves as a predictor of postoperative hemodynamic improvement.

**Patients and methods:**

We prospectively included patients with symptomatic cerebrovascular steno-occlusive disease who underwent STA-MCA bypass with pre- and postoperative BOLD-CVR imaging and intraoperative bypass flow measurements. Pearson correlation and multivariable regression models assessed the relationships between preoperative hemodynamic status (i.e. preoperative BOLD-CVR), intraoperative bypass flow, and postoperative BOLD-CVR improvement, adjusting for confounders (type of steno-occlusive disease, age, and cerebrovascular risk factors).

**Results:**

Forty-three patients (three receiving bilateral bypass) were included. Despite lack of association (*p* = 0.08) at univariable analysis, multivariable regression analysis revealed that, after correction for known confounders, preoperative CVR in the affected MCA territory was inversely associated with intraoperative bypass flow. For each 0.1 unit (percentage BOLD signal change/mmHg CO2) decrease in preoperative MCA territory CVR, the predicted bypass flow increased by 14.61 mL/min. Preoperative CVR was also the only significant predictor of postoperative CVR, with higher preoperative BOLD-CVR values linked to greater hemodynamic improvement.

**Conclusion:**

The severity of preoperative hemodynamic impairment in the affected MCA territory correlates with the increased need for bypass flow, serving as a potential predictor for intraoperative quantitative bypass flow demand once relevant covariates are accounted for. The STA-MCA bypass appears to deliver optimal flow when the cerebrovascular reserve capacity is not fully exhausted.

## Graphical abstract

**Graphical abstract:**
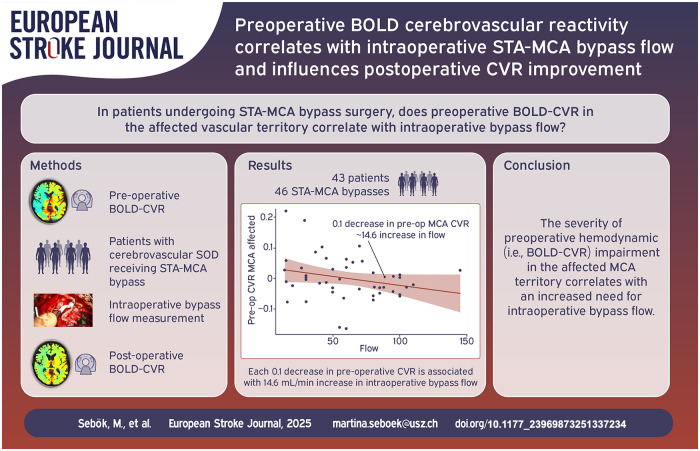

## Introduction

Surgical revascularization via a flow augmentation superficial temporal artery-middle cerebral artery (STA-MCA) bypass has been proven an effective treatment for decreasing the risk of recurrent ischemia in patients with Moyamoya vasculopathy and selected cases of atherosclerotic large vessel occlusion despite negative results of randomized trials performed in last decades.^1–5^ Although the STA-MCA bypass is traditionally considered a low-flow bypass, our recent study of intraoperative and postoperative quantitative flow measurements revealed that the degree of flow augmentation in the bypass conduit is influenced by the demand of the revascularized territory. Flow demand can reach higher levels (up to 145 mL/min) when required, with the bypass capacity increasing over time.^6^

In patients with symptomatic cerebrovascular steno-occlusive disease, in general, the cerebral perfusion pressure to the affected cerebral hemisphere is decreased^7,8^ which can be assessed as impaired cerebrovascular reactivity (CVR).^7,9–11^ In more severe cases, upon hypercapnic vasodilatory stimulation, a paradoxical – negative – BOLD signal response is observed (i.e. steal phenomenon) due to the impossibility of vessels with exhausted cerebrovascular reserve to further dilate, with consequential blood flow redistribution away from the suffering brain tissue (which then suffers an hemodynamic steal in stress condition).^12^ Previous studies have shown the effectiveness of bypass revascularization using CVR testing and showed that the hemodynamics of the revascularized hemisphere improve following the bypass surgery.^13–15^ Furthermore, the hemodynamic improvement has also been observed in the non-revascularized hemisphere.^16^

Blood oxygenation-level dependent cerebrovascular reactivity (BOLD-CVR) is increasingly used to analyze hemodynamic impairment, thereby aiding the identification which patients exhibit a higher risk for recurrent ischemic events.^10,15,17^ In combination with a carbon dioxide (CO_2_) stimulus, cerebrovascular reactivity can be measured.^18,19^ Using a calibrated measurement technique with a CO_2_ stimulus, this allows for longitudinal systematic evaluation of patients with cerebrovascular steno-occlusive diseases and to select candidates for flow augmentation revascularization surgery.^15,20^ However, the relationship between preoperative CVR impairment and intraoperative quantitative STA-MCA bypass flow has yet to be studied. Furthermore, monitoring pre- and postoperative cerebrovascular reactivity allows us to assess the relationship between intraoperative bypass flow and subsequent hemodynamic improvement.

In this study, we aimed to analyze the relationship between pre- and postoperative BOLD-CVR and intraoperative quantitative bypass flow by testing the following two hypotheses: (1) Lower preoperative BOLD-CVR values in the affected MCA territory lead to higher intraoperative STA-MCA bypass flow, and (2) Higher intraoperative STA-MCA bypass flow leads to better BOLD-CVR improvement in the affected MCA territory.

## Materials and methods

### Patient selection

We included all patients with symptomatic cerebrovascular steno-occlusive disease who underwent microsurgical STA-MCA flow augmentation bypass at the Department of Neurosurgery, University Hospital Zurich, Switzerland, between January 2015 and July 2024. Eligible patients underwent preoperative and postoperative BOLD-CVR studies and intraoperative quantitative bypass flow measurement using the Charbel Flowprobe^®^. Clinical data were extracted from a prospectively collected institutional registry, approved by the local ethics review board and registered at ClinicalTrials.gov (identifier NCT01628406). The research ethics board of Canton Zurich (KEK-ZH-Nr. 2012-0427 & KEK-ZH-Nr. 2020–02314) approved the BOLD-CVR imaging study conducted at the same institution.

### Image acquisition and processing

MRI data were collected on a 3-tesla Skyra VD13 scanner (Siemens Healthcare, Erlangen, Germany) equipped with a 32-channel head coil. BOLD fMRI settings and a 3D T1-weighted Magnetization Prepared Rapid Acquisition Gradient Echo (MP-RAGE) sequence were applied following the protocol detailed in our previous studies.^21^ During the fMRI sequence, a computer-controlled gas blender with prospective gas targeting algorithms (RespirAct, Thornhill Research Institute, Toronto, Canada) modulated the carbon dioxide stimulus. The RespirAct system enables precise control of arterial partial pressures of oxygen and carbon dioxide.^22^ A controlled hypercapnic stimulus was administered to patients during the BOLD-CVR study, as previously described.^23^ All raw BOLD MRI volumes were transferred to an external computer and pre-processed using SPM 12 (Statistical Parametric Mapping, Wellcome Department of Imaging Neuroscience, University College London, London, UK). The BOLD volumes were aligned to the T1-weighted MP-RAGE image, normalized to Montreal Neurological Institute (MNI) space, and smoothed with a Gaussian kernel.^21^

#### BOLD-CVR

CVR analysis was performed in MATLAB by means of in-house written scripts using a previously established pipeline, including voxel-wise temporal shifting for optimal correlation between the BOLD signal and CO2 time series. CVR, defined as the percentage BOLD signal change per mmHg CO2, was calculated from the slope of the linear fit between the BOLD signal and CO2 time series during the first 100-second baseline, the 80–second step portion, and the second 100–second baseline. Extra BOLD MRI volumes were acquired to account for potential temporal shifts.^24^

#### Middle cerebral artery territory analysis

BOLD-CVR values for the MCA territory in the affected hemisphere were obtained using a vascular atlas based on predefined brain regions from the standard N30R83 atlas by Hammers et al.^25^ and Kuhn et al.^26^ to the normalized CVR maps.

### Intraoperative quantitative bypass flow measurement

Using a flexible perivascular Charbel transit-time flow probe (Charbel Micro-Flowprobe^®^, Transonic Systems Inc.), we measured the flow in the bypass intraoperatively in milliliters per minute as described previously.^6^

### BOLD-CVR follow-up study

As standard practice at our institution, all patients underwent a postoperative BOLD-CVR study approximately 3 months after bypass surgery to assess brain hemodynamic changes following revascularization.

### Statistical analysis

Statistical analysis was conducted using R Studio v.2024.9.0.375 (Posit team, 2024). Normally distributed continuous variables are reported as mean ± SD, categorical ordinal variables as median (IQR), and dichotomous variables as frequency (%). The Shapiro test assessed normality. Pre- and postoperative CVR values were compared using paired t-tests for normal data and Wilcoxon signed-rank tests for non-normal data, with significance set at α = 0.05. Pearson correlation examined relationships between preoperative CVR, intraoperative flow, and postoperative CVR or delta CVR.

Univariable linear regression analyzed preoperative variables’ impact on bypass flow and the relationships of preoperative and intraoperative variables with postoperative CVR. Two multiple linear regression models tested research hypotheses: (1) preoperative MCA-CVR’s effect on bypass flow, adjusted for demographic and clinical factors, and (2) intraoperative flow’s effect on postoperative CVR, adjusted for pre-operative CVR and significant confounders. Models included baseline factors, excluded aliased coefficients and redundant predictors, and evaluated multicollinearity (variance inflation factor > 5 removed). Stepwise selection with Akaike Information Criterion optimized model fit, reintroducing significant univariable variables or confounders if necessary (10 subjects/predictor rule). Statistical significance was set at *p* < 0.05.

## Results

### Study population characteristics

Between January 2015 and July 2024, 43 patients underwent preoperative BOLD-CVR assessment before undergoing flow augmentation STA-MCA bypass surgery with intraoperative flow measurements. Among them, three patients later received contralateral bypasses, resulting in a total of 46 bypasses. Follow-up BOLD-CVR assessments were conducted approximately 3 months post-surgery (Supplemental Figure 1). The mean interval between preoperative CVR and surgery was 14 days (SD 70). Sixteen patients (37.2%) had Moyamoya disease, nine (56.3%) unilateral and seven (43.7%) bilateral, with three undergoing bilateral bypasses. Fifteen patients (34.9%) had chronic ICA occlusion with recurrent symptoms, eight (18.6%) had acute ICA occlusion, and four (9.3%) had MCA occlusion (two acute, two chronic). Baseline cohort characteristics are detailed in Supplemental Table 1.

### Impact of surgical revascularization on BOLD cerebrovascular reactivity

**Figure 1.**
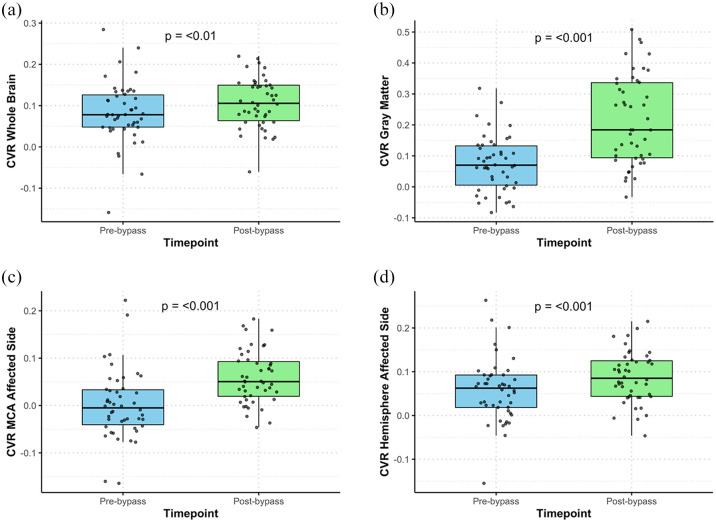
Box plots comparing cerebrovascular reactivity of selected volumes-of-interest (whole brain, gray matter, affected MCA territory, affected hemisphere) before and after STA-MCA bypass revascularization. (a) CVR whole brain pre-bypass versus post-bypass, (b) CVR gray matter pre-bypass versus post-bypass, (c) CVR affected MCA pre-bypass versus post-bypass, and (d) CVR affected hemisphere pre-bypass versus post-bypass. Significant increases in BOLD-CVR values after STA-MCA bypasses are shown for all displayed volumes-of-interest. Each boxplot includes individual data points (black dots) and represents the median, interquartile range, and variability in CVR values at the two time points (before and after STA-MCA bypass surgery).

The mean baseline end tidal oxygen pressure (PETO_2_) and end tidal carbon dioxide pressure (PETCO_2_) during the hypercapnic stimulus were consistent across preoperative and postoperative BOLD-CVR studies, indicating optimal technical execution with no significant variability (Supplemental Table 2). Postoperative CVR significantly improved compared to preoperative values across the whole brain, whole brain gray matter, the affected hemisphere, and the affected MCA and ACA vascular territories while no statistically significant improvement was observed in whole brain white matter, unaffected ACA and MCA territory and PCA territory bilaterally (Figure 1, Supplemental Table 2).

### Correlation between intraoperative bypass flow and BOLD-CVR values

Pearson correlation assessed relationships between: (1) preoperative CVR in the MCA territory and intraoperative bypass flow (*r* = −0.25, 95% CI −0.51 to 0.03, *p* = 0.08), (2) intraoperative bypass flow and postoperative CVR in the MCA territory (*r* = −0.33, 95% CI −0.57 to −0.05, *p* = 0.02), and (3) intraoperative flow and pre/to/postoperative change of CVR in the MCA territory (*r* = −0.0002, 95% CI −0.29 to 0.29, *p* = 0.99).

Simple linear regression explored clinical variables and risk factors affecting intraoperative bypass flow (Supplemental Table 3) and postoperative CVR in the MCA territory (Supplemental Table 4). While no variables significantly predicted intraoperative flow, preoperative CVR in the MCA territory showed an association, despite not reaching statistical significance (*p* = 0.08). For postoperative CVR in the MCA territory, significant predictors included intraoperative bypass flow (*p* < 0.05), preoperative CVR (*p* < 0.001), hypertension (*p* < 0.05), and smoking (*p* < 0.05).

### Flow and CVR prediction models

Two linear regression models were constructed to assess: (1) the impact of preoperative MCA territory CVR on intraoperative bypass flow and (2) whether intraoperative bypass flow predicts improvements in postoperative MCA territory CVR. Aliased coefficients identified “steno-occlusive disease acute” as redundant due to its predictability from “steno-occlusive disease chronic” and “moyamoya,” leading to its exclusion. Variance inflation factor analysis showed no multicollinearity. Stepwise selection identified key predictors, with confounders reintroduced due to the small number of retained variables.

#### Model 1: Intraoperative Bypass flow

Predictors: preoperative MCA-CVR, chronic steno-occlusive disease, age, hypertension, and moyamoya disease.

**Figure 2.**
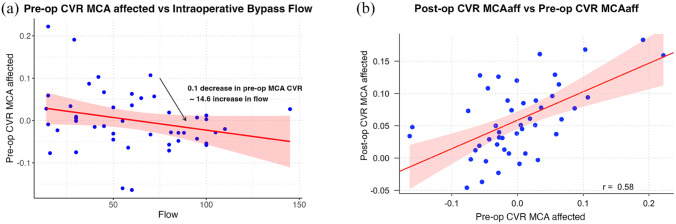
Relationship between preoperative CVR in the affected middle cerebral artery territory and intraoperative bypass flow (Model 1), and association between preoperative and postoperative CVR in the affected MCA territory (Model 2). Scatterplot (a) shows the relationship between preoperative CVR in the affected MCA and intraoperative bypass flow. The red line represents the fitted regression line with a shaded 95% confidence interval. A decrease of 0.1 in preoperative CVR (percentage BOLD signal change/mmHg CO2) is associated with an approximate increase of 14.6 ml/min in bypass flow, indicating an inverse relationship. Scatterplot (b) illustrates the association between preoperative CVR and postoperative CVR in the affected MCA. The positive correlation (r = 0.58) is indicated by the fitted regression line (red) with a 95% confidence interval (shaded area), suggesting that higher preoperative CVR is associated with higher postoperative CVR.

Preoperative MCA-CVR (coefficient: −146.1, *p* < 0.05) was the only significant predictor, indicating an inverse relationship (Table 1). A 1-unit decrease in preoperative MCA-CVR predicts a 146.1 mL/min increase in intraoperative flow, or approximately 14.61 mL/min for a 0.1-unit decrease (Figure 2(a)).

**Table 1.** Multivariable linear models for prediction of intra-operative bypass flow (model 1) and post-operative CVR (model 2).

Model 1				
*N* = 46				
Dependent Variable: Intra-operative flow				
	Est	S.E.	t.val.	*p*-Value
Intercept	90.093	30.717	2.933	0.006
Pre-operative CVR	−146.096	67.842	−2.153	<0.05
Chronic SOD	−19.442	12.281	−1.583	0.121
Age	−0.224	0.495	−0.454	0.653
MoyaMoya	−11.16	13.424	−0.831	0.411
Hypertension	−6.935	11.946	−0.581	0.565
Model 2				
*N* = 46				
Dependent Variable: Post-operative CVR				
	Est	S.E.	t.val.	*p-*Value
Intercept	0.085	0.038	2.24	0.031
Pre-operative CVR	0.339	0.105	3.219	<0.01
Flow	0	0	−1.649	0.107
Age	0	0.001	0.5	0.62
Hypertension	−0.02	0.017	−1.171	0.249
Smoking	−0.018	0.011	−1.615	0.114

CVR: cerebrovascular reactivity; *N*: number; SOD: steno-occlusive disease.

Estimated coefficients (Est), standard errors (S.E.), t-values (t.val.), and *p*-values are reported.

#### Model 2: Postoperative MCA-CVR

Predictors: intraoperative flow, preoperative MCA-CVR, age, hypertension, and smoking.

Preoperative MCA-CVR (coefficient: 0.339, *p* < 0.01) was the only significant predictor, with a 1-unit increase in preoperative MCA-CVR predicting a 0.339-unit increase in postoperative MCA-CVR (Table 1). On the 0.1 CVR scale, this translates to a 0.033-unit increase per 0.1-unit preoperative rise. Intraoperative flow and other variables showed no significant associations (Figure 2(b)).

## Discussion

Using advanced BOLD-CVR and intraoperative volumetric flow measurements in patients with symptomatic cerebrovascular steno-occlusive disease, we aimed to determine whether preoperative CVR impairment predicts intraoperative bypass flow and to assess how intraoperative flow influences postoperative CVR outcomes. In multivariable linear regression, preoperative CVR was the only factor inversely associated with intraoperative bypass flow. Specifically, each 0.1 -unit decrease in preoperative CVR (percentage BOLD signal change/mmHg CO2) was linked to a 14.61 mL/min increase in intraoperative bypass flow. Additionally, the preoperative CVR was the sole predictor of postoperative CVR, highlighting the value of timely surgical intervention before CVR exhaustion to allow for greater postoperative CVR improvements.

### Impact of surgical revascularization via STA-MCA bypass on brain hemodynamic status assessed by BOLD-CVR

Our data corroborate previous reports^11–14,17^ that STA-MCA bypass significantly improves cerebrovascular reactivity in the revascularized hemisphere, with the greatest improvement observed in the MCA territory, and with the degree of CVR improvement correlated to the initial severity of hemodynamic impairment.^11^ As an inadequate CVR is regarded a major risk factor for subsequent cerebral infarction,^27–29^ a proper identification of patients with impaired hemodynamic who could benefit from revascularization plays a crucial role in the treatment of those patients.^10,30^

### Predictive value of preoperative BOLD-CVR impairment on intraoperative STA-MCA bypass flow

This study systematically evaluated pre- and postoperative hemodynamic status and its relationship with intraoperative bypass flow to test the two hypotheses. Although not statistically significant in univariate analysis, multivariable linear regression, after adjusting for confounders, identified preoperative CVR as the only factor inversely associated with intraoperative bypass flow. A 0.1-unit decrease in preoperative CVR (% BOLD signal change/mmHg CO2) was linked to a 14.61 mL/min increase in bypass flow. The ability of impaired preoperative BOLD-CVR to predict bypass flow offers the following key advantages: (1) optimized patient selection by identifying those most likely to benefit from bypass surgery, (2) refined surgical planning by anticipating required flow and guiding vessel selection, (3) enhanced outcome prediction, (4) personalized treatment tailored to the patient’s hemodynamic profile, and (5) improved postoperative monitoring, particularly for hyperperfusion^31^ risks in high-flow cases.

Interestingly, higher intraoperative bypass flow does not correlate with improved CVR at 3 months and instead shows a negative association. Nevertheless, CVR significantly improves within the revascularized vascular territory, as previously demonstrated.^15,20^ These findings suggest that hemodynamically compromised tissue demands additional flow to meet its metabolic and vascular needs. Univariable and multivariable linear analyses further explored these relationships. After adjusting for confounders, multivariable analysis identified preoperative CVR as the sole predictor of intraoperative bypass flow. This reinforces the correlation and indicates that greater preoperative vascular (i.e. hemodynamic) compromise is linked to increased flow demands from the affected brain tissue.

### Correlation between preoperative and postoperative BOLD-CVR

No correlation was found between intraoperative bypass flow and postoperative CVR in the multivariable analysis. After adjusting for confounders, preoperative CVR was the only factor associated with postoperative CVR, with higher preoperative BOLD-CVR values linked to greater hemodynamic improvement. This suggests that EC-IC bypass is most beneficial when some cerebrovascular reserve is preserved preoperatively, allowing for compensatory mechanisms. Our models show that lower preoperative CVR still leads to positive postoperative change, though less pronounced, while higher preoperative CVR results in greater and more immediate improvement. These findings highlight the diagnostic and prognostic value of BOLD-CVR in assessing hemodynamics and guiding patient selection and timing for surgical revascularization.

### Strengths of the study and future outlooks

This study presents the first cohort of patients undergoing STA-MCA bypass with pre- and postoperative hemodynamic assessments and intraoperative quantitative flow measurements. Impaired CVR is a well-known risk factor for recurrent ischemic stroke.^29^ This study highlights the importance of initial hemodynamic assessment in patients with steno-occlusive disease undergoing surgical revascularization. Our findings show that preoperative CVR significantly impacts intraoperative bypass flow and postoperative CVR improvement. Future research should explore whether higher bypass flow reduces stroke recurrence by enhancing long-term CVR. If so, optimizing anastomosis size for greater flow in high-demand territories may be beneficial, informing the optimal timing for EC-IC bypass in this population.

### Limitations

Despite robust statistical methods, some confounders may have been missed due to the small sample size, though this remains a large, single-center study of a complex patient population. The single-center design limits generalizability, necessitating multicenter validation. Variations in pre- and postoperative BOLD-CVR assessments, based on clinical scenarios and surgeon preferences, may impact results. While intraoperative bypass flow measurements were accurate, they may change over time, and future studies should investigate the relationship between long-term bypass flow, longitudinal CVR assessment at later timepoints, and possible hemodynamic improvement extending also to the contralateral hemisphere which was not observed in the present investigation.^16,20^ Furthermore, we included a mixed cohort of patients with atherosclerotic large vessel occlusion and Moyamoya vasculopathy, as our goal was not to study disease pathophysiology but rather to explore and assess the relationship and predictive value of CVR and bypass flow.

## Conclusions

The severity of preoperative cerebrovascular reactivity impairment correlates with the demand for increased bypass flow, serving as a potential predictor for intraoperative quantitative bypass flow magnitude. The STA-MCA bypass appears to be optimal when the cerebrovascular reserve is not fully exhausted, highlighting the importance of timely intervention.

## Supplemental Material

sj-pdf-1-eso-10.1177_23969873251337234 – Supplemental material for Preoperative BOLD cerebrovascular reactivity correlates with intraoperative STA-MCA bypass flow and influences postoperative CVR improvement Supplemental material, sj-pdf-1-eso-10.1177_23969873251337234 for Preoperative BOLD cerebrovascular reactivity correlates with intraoperative STA-MCA bypass flow and influences postoperative CVR improvement by Martina Sebök, Vittorio Stumpo, Jacopo Bellomo, Giuseppe Esposito, Christiaan Hendrik Bas van Niftrik, Zsolt Kulcsár, Andreas R. Luft, Luca Regli and Jorn Fierstra in European Stroke Journal
