# Supplementary material for: Preoperative BOLD cerebrovascular reactivity correlates with intraoperative STA-MCA bypass flow and influences postoperative CVR improvement
Source: Eur Stroke J. 2025 May 10;10(4):1454–61. doi: 10.1177/23969873251337234 (PMC12065708; doi:10.1177/23969873251337234)
Supplement: sj-pdf-1-eso-10.1177_23969873251337234 – Supplemental material for Preoperative BOLD cerebrovascular reactivity correlates with intraoperative STA-MCA bypass flow and influences postoperative CVR improvement [file sj-pdf-1-eso-10.1177_23969873251337234.pdf]

Supplementary Tables

Supplementary Table 1. Baseline characteristics of the study cohort

	**Mean (SD)**
**Age**	58.43 (12.72)
**Pathology**	**n (%)**
**Moyamoya**	16 (37.2%)
Unilateral	7 (43.7%)
Bilateral	9 (56.3%)
**ICA chronic occlusion**	15 (34.9%)
**ICA acute occlusion**	8 (16.6%)
**MCA acute occlusion**	2 (4.6%)
**MCA chronic occlusion**	2 (4.6%)
	**n (%)**
**Female sex**	17 (36.9%)
**Cerebrovascular risk**	37 (80.4%)
**Hypertension**	29 (63.0%)
**Diabetes**	11 (23.9%)
**Dyslipidemia**	29 (63.0%)
**Smoking**	21 (45.6%)
**Stroke**	22 (47.8%)
ICA=internal carotid artery, MCA=middle cerebral artery, N=number, SD=standard deviation	

Supplementary Table 2. Baseline and 3-month follow-up BOLD-CVR data for the entire study cohort

	**Pre-bypass**	**Post-bypass**	**p-value**
**$PETCO_2$ (mmHg)**			
**Mean Baseline**	39.31 (4.34)	39.02 (4.29)	0.75
**Mean Step**	48.54 (4.50)	48.28 (4.57)	0.78
**Stepchange**	9.23 (1.06)	9.26 (1.02)	0.92
**BOLD-CVR (%BOLD signal change/mmHg CO_2)**			
**Whole brain**	**0.08 (0.07)**	**0.11 (0.06)**	**<0.01****
**Gray matter**	**0.07 (0.09)**	**0.22 (0.14)**	**<0.001*****
**White matter**	0.06 (0.06)	0.06 (0.05)	0.67
**Affected hemisphere**	**0.06 (0.07)**	**0.09 (0.06)**	**<0.001*****
**Unaffected hemisphere**	0.11 (0.08)	0.08 (0.06)	0.085
**ACA affected**	**0.04 (0.07)**	**0.06 (0.06)**	**<0.01****
**ACA unaffected**	0.07 (0.07)	0.09 (0.07)	0.08
**MCA affected**	**0.00 (0.07)**	**0.06 (0.06)**	**<0.001 *****
**MCA unaffected**	0.08 (0.09)	0.09 (0.07)	0.18
**PCA affected**	0.17 (0.08)	0.17 (0.07)	0.542
**PCA unaffected**	0.18 (0.09)	0.18 (0.07)	0.77

ACA=anterior cerebral artery, BOLD=blood oxygenation-level dependent, CVR=cerebrovascular reactivity, MCA=middle cerebral artery, PCA=posterior cerebral artery
Normality was tested using Shapiro test. Wilcoxon-signed rank test for non-normally distributed data. Two-tailed paired t-test was used for normally distributed data.

Supplementary Table 3. Simple linear regression explored clinical variables and risk factors affecting intraoperative bypass flow

Dependent variable	Intra-operative STA-MCA Bypass Flow				
**Independent variable**	**Coefficient**	**SE**	**T value**	**P value**	**Rsquared**
Age	-0.2121096	0.36948599	-0.5740668	0.57	0.007
Sex	3.81338742	9.64959288	0.39518635	0.69	0.004
Hypertension	-4.7583333	9.77043305	-0.4870136	0.63	0.005
Smoking	5.22347066	7.54278268	0.69251242	0.49	0.011
Dyslipidemia	5.61486486	8.78076416	0.63945059	0.53	0.009
Diabetes mellitus	7.9754902	10.5578043	0.75541182	0.45	0.013
Stroke	-3.6260409	5.88005975	-0.6166674	0.54	0.009
**Pre-operative MCA CVR**	**-112.25694**	**63.1155663**	**-1.7785935**	**0.08**	**0.067**
Moyamoya	3.68421053	9.46010408	0.38944715	0.70	0.003
Chronic SOD	-14.077079	9.43087537	-1.4926588	0.14	0.048
Acute SOD	14.0277778	11.1128614	1.26230115	0.21	0.035

Abbreviations: CVR, cerebrovascular reactivity; MCA-CVR, middle cerebral artery; SE, standard error; SOD, steno-occlusive disease; STA-MCA, superficial temporal artery-middle cerebral artery

Supplementary Table 4. Simple linear regression explored clinical variables and risk factors affecting postoperative CVR in the MCA territory

Dependent variable	Post-operative CVR MCA territory				
**Independent variable**	**Coefficient**	**SE**	**T value**	**P value**	**Rsquared**
**Flow**	**-0.0006**	**0.000251**	**-2.37937**	**<0.05**	**0.114**
Age	-0.00058	0.00065	-0.89785	0.37	0.017992
Sex	-0.02088	0.016816	-1.24147	0.22	0.033843
**Hypertension**	**-0.0338**	**0.016572**	**-2.03956**	**<0.05**	**0.086375**
**Smoking**	**-0.0267**	**0.012804**	**-2.08526**	**<0.05**	**0.089937**
Dyslipidemia	-0.02654	0.015091	-1.75852	0.08	0.065666
Diabete mellitus	-0.02057	0.018548	-1.1092	0.27	0.027201
Stroke	-0.00055	0.010451	-0.05244	0.96	6.25E-05
**Pre-operative MCA CVR**	**0.441473**	**0.094574**	**4.667994**	**<0.001**	**0.331207**
Moyamoya	0.015497	0.016607	0.93314	0.35	0.019406
Chronic SOD	-0.01057	0.017033	-0.62042	0.54	0.008672
Acute SOD	-0.00761	0.019987	-0.3808	0.70	0.003285

Abbreviations: CVR, cerebrovascular reactivity; MCA-CVR, middle cerebral artery; SE, standard error; SOD, steno-occlusive disease; STA-MCA, superficial temporal artery-middle cerebral artery

Supplementary Figures

Supplementary Figure 1: Study Flowchart

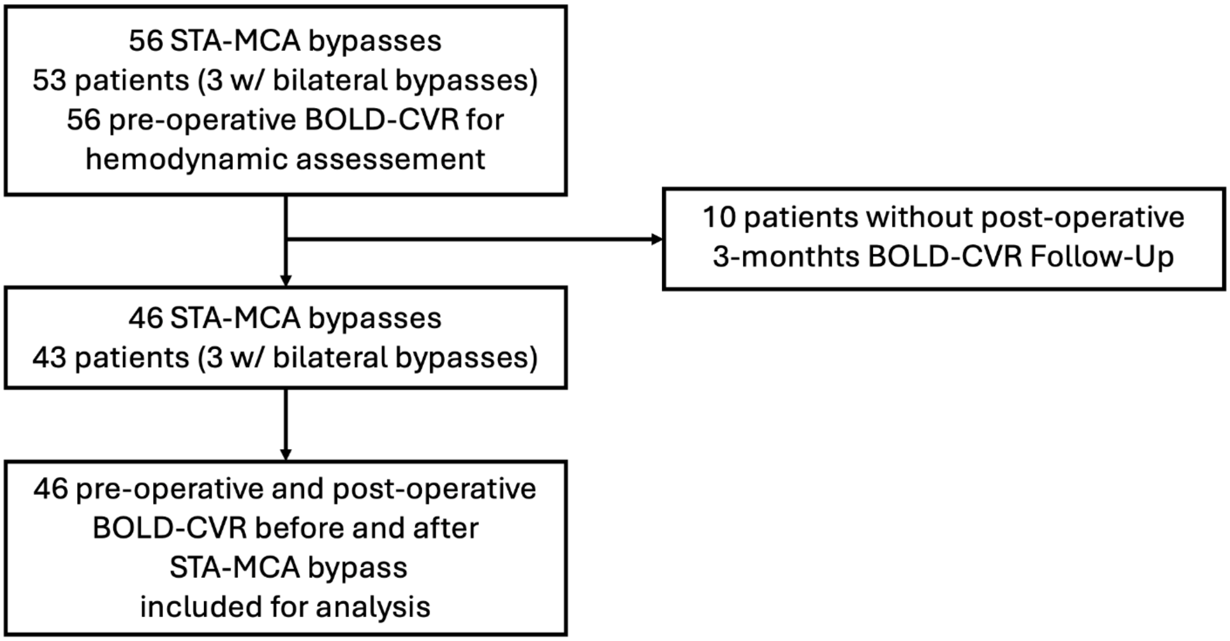

Abbreviations: BOLD=blood oxygenation-level dependent, CVR=cerebrovascular reactivity, MCA=middle cerebral artery, STA=superficial temporal artery

Supplementary Figure 2: Pre- and Postoperative BOLD-CVR Maps of an Exemplary Patient

Caption: This illustrative case depicts a patient with preoperative exhausted cerebrovascular reserve capacity in the right hemisphere due to right internal carotid artery occlusion. At the 3-month follow-up after surgical revascularization via a right STA-MCA bypass, cerebrovascular reactivity shows notable improvement in the revascularized territory.

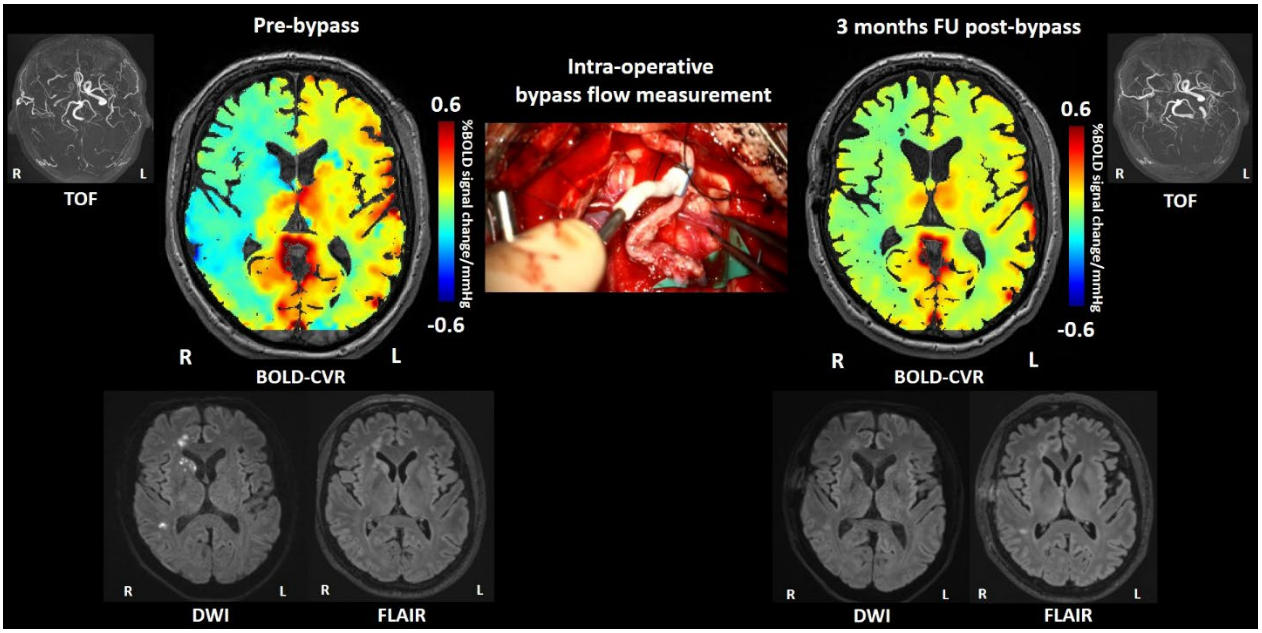

Abbreviations: BOLD=blood oxygenation-level dependent, CVR=cerebrovascular reactivity, DWI =diffusion-weighted imaging, FLAIR=fluid-attenuated inversion recovery, TOF=time-of-flight
